# Eye-Hand Synchronization in Xylophone Performance: Two Case-Studies with African and Western Percussionists

**DOI:** 10.16910/jemr.11.2.7

**Published:** 2019-03-31

**Authors:** Fabrice Marandola

**Affiliations:** McGill University, Montreal, Canada; Museum National d’Histoire Naturelle, Paris, France

**Keywords:** Eye movement, eye tracking, gaze, field research, eye-strokespan, eye-hand span, percussion, xylophone, individual differences

## Abstract

This article is the result of a first foray into xylophone performance with percussionists from Canada and Cameroon. It proposes to use the combination of Eye-Stroke Span (ESS), Fixation- Duration and Note-Pattern indexes to analyze free-score and performance oriented musical tasks, instead of eye-hand span or awareness span for sight-reading and score-based eye-tracking research in music. Based on measurements realized with a head-mounted eyetracker system, the research examines gaze-movements related to eye-hand synchronization in xylophone performance with musicians coming from three different ethnic groups from Cameroon (Bedzan Pygmies, Tikar and Eton) and classically trained Western percussionists (Canada). Increases in tempo are found to involve a diminution of the number of fixations, but not proportionally, as well as changes in lateral gaze shifts. Fixation-Duration and Note- Pattern are closely related but not identical, while ESS is relatively more independent. These gaze patterns are consistent within individuals, but not across individuals. Cameroonian musicians tend to look away from their instrument, interacting with their peers or with the audience. When they look at their keyboard, preliminary measures of ESS were found similar to the ESS of Western performers.

## Introduction

Xylophones are percussion instruments made of a series of tuned wooden bars of varying length, width and thickness (the ‘keyboard’). They are played by performers who use mallets to strike the bars (xylophones, marimbas, vibraphones and other pitched instruments are often referred to as ‘keyboard percussion’ or ‘mallet instruments’ in Western classical music). A common way to organize the keyboard is from low to high range (modern Western xylophones are a good example) but other configurations can be found around the world (for a review on xylophones, see (1)). Some xylophones have resonators mounted below the bars to enhance sound quality, resonance and sometimes tuning (2), but a common feature of xylophones is to produce sounds that are relatively short, especially in the high range (3). As a result, in most cultures, xylophone repertoire tends to privilege music at fast tempos where many notes are played in quick succession, and being able to play at high speeds with great accuracy is the hallmark of excellent performers: a good example in the Western tradition can be found in the numerous recordings of xylophone virtuoso George Hamilton Green, dating from the first half of the 20th Century (4).

During xylophone performance, the only contact between the performer and the keyboard is indirect and happens during the very brief moment of impact between the head of the mallet and the bar. Contrary to the practice of instruments such as the violin for example, where haptic feedback is rich and integral to the interaction between instrument and performer (5), xylophone performance provides a reduced amount of haptic feedback. The sense of sight is thus particularly solicited during training phases and performance, and the capacity to visualize the notes to be hit is a topic often raised during discussions among peers, as well as in the teaching studios of Western practitioners (6). Moreover, percussionists have to work with instruments whose shapes are asymmetric: bars differ in width and length between low and high ranges (in the low register of a marimba, which could be described as a bass xylophone, the bars may be twice as wide in the low range as in the highest range), and between models (even in the world of Western classical music where instruments are mass-produced, xylophone dimensions vary from one brand to another). Therefore, not only must percussionists accurately hit many different targets at high-speeds in a pre-set order (the different pitches that constitute the melody that they play), they must also adapt to the built-in variability that is inherent to the nature of their instrument.

Stemming from over 25 years of observation and practice as a professional percussionist and teacher of Western classical music, as well as an ethnomusicologist researching music from Central African Republic and Cameroon, the main question behind this research is fairly simple: where do performers look while they are playing the xylophone?

Internationally acclaimed marimba soloist Leigh Howard Stevens stresses the importance of vision and the role of anticipation in performance: “For me, the most important thing is visualizing what notes I’m going to hit” ((6) p. 37). This observation illustrates the well-known role of the anticipation of the gaze in many motor-control tasks (7) and leads us to focus our research question: how do xylophone players anticipate the action of their hands with their gaze while they are performing? In other terms, how are the eyes and hands synchronized in xylophone performance? Two related questions stem from this main interrogation: do all percussionists anticipate with their gaze in the same way? And what relationship exists between performance speed and gaze anticipation?

Research on music and eye-movement is largely based on experiments where musicians read the music they are playing, the experimental conditions varying between sight-reading and different levels of rehearsed performances. When the music is performed from a score, musicians tend to look ahead to the next notes to be played: the distance between the note being played and the note that is fixated upon by the eye is called Eye-Hand Span (8), or EHS. EHS can be denoted in duration (typically in milliseconds) or by the number of notes between hand and eye position, and depends on multiple factors such as the performance speed, the level of expertise of the performer or the complexity of the music to be performed (9, 10, 11, 12). The notion of awareness span (13) was introduced to measure the adaptation of EHS before a glance at the keyboard of the piano, taking into account the moments when the player needs to look at the keyboard a few milliseconds after reading the score, in order to perform accurately. This measurement helps to understand how pianists navigate between two systems of reference: the code that represents a musical score, and its application on the physical plane via the keyboard of the piano. However, when music is learnt by memory, EHS becomes less relevant since there is a ‘more-or-less complete dissociation of the eye movements from the written music […], presumably because the buffer is now obtaining its input form some other source, and the eye movements are no longer relevant’ (9). In other terms, the eye movements of musicians playing by memory are no longer driven by the necessity to refer to a physical musical score (paper or electronic device), and are thus focused differently (on the instrument or the environment).

**Figure 1.**
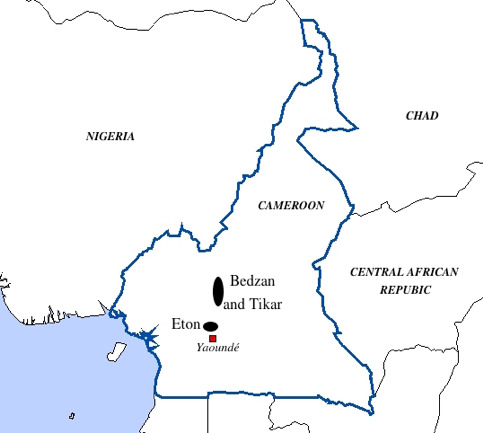
Map of Cameroon and localization of the three ethnic groups involved in this study. Adapted from <johan.lemarchand.free.fr>

Most of the research on anticipation, memorization and eye-movement in music is indeed based on music played from a score, and music performance in score-free conditions has seldom been studied. Moreover, xylophone performance has never been explored using eye-tracking devices: we do not know where xylophonists look when they perform, and what strategies they use during their performances to ensure the best accuracy. This article presents preliminary results from a larger interdisciplinary project dedicated to the study of instrumental gesture (14). It investigates how a xylophonist’s gaze moves during a performance, focusing on score-free musical tasks. Two contrasting contexts are examined in separate case-studies: classically-trained Western performers from the conservatory and university systems in Canada forming one group, and musicians belonging to three different ethnic groups from Cameroon (Bedzan Pygmies, Tikar and Eton; Figure 1), trained according to oral tradition by immersion, observation and imitation (15) in the other group.

It may seem paradoxical to lead a score-free study with classically trained musicians, who spend a large part of their musical practice reading musical notation and playing from a score; however, xylophone training is largely based (as is the case with many other instruments) on exercises and routines that are entirely performed by memory. In most cases performers also play their pieces by memory, and this is particularly true for orchestral excerpts, which are short passages from the orchestral repertoire that are used to test and select performers during professional auditions: playing by memory allows musicians to look at their keyboard in order to ensure the greatest accuracy throughout their performance.

## Method

**Figure 2.**
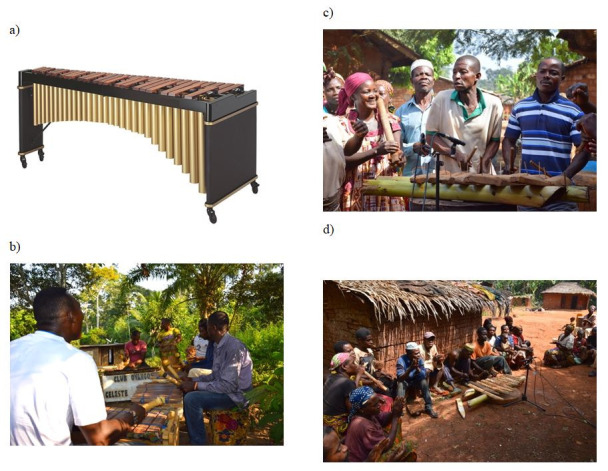
The different types of xylophones studied: a) Western marimba; b) ensemble of xylophones with multiple resonators – Eton, village of Emana, Cameroon; c) banana-trunk xylophone played in a stand-up position - Tikar, village of Beng-Beng, Cameroon; d) banana-trunk xylophone placed on the ground - Bedzan, village of Mansoh, Cameroon. Photo credit: F. Marandola (2015)

For the first case-study, the measurements took place in Canada in two phases: the first phase involved 9 percussionists and involved a series of technical exercises based on scales, typical of Western musical training practices, as well as a musical excerpt from standard orchestral xylophone repertoire, recorded at the Centre for Interdisciplinary Research in Music, Media and Technology (CIRMMT, Montreal). For each task, musicians were asked to perform at different speeds to evaluate the effect of speed on eye-hand synchronization. In sight-reading tasks, different methods were used regarding the choice of tempo: self-paced, fixed by a metronome or a recorded accompaniment, or a combination of both. The latter method was used by Lehmann and Ericsson (16) who used trials with fixed tempo to measure differences in performance, but noted that the reasons behind the selection of tempo in self-paced trials were varying and not always clearly stated, and that variations in tempo could occur "without violating the range of acceptable tempi for performance" (p. 193). In the first phase of my research, xylophonists chose their own performance speed and played an excerpt of their choice: the freedom in the choice of performance speed aimed to collect data relative to potential thresholds linked to speed that would be dependent on individual skills. The second phase was run in the faculty’s percussion studio, with 3 musicians, one of them having already taken part in the first set of experiments but whose measurements were not of sufficient quality (high data loss rate). In order to facilitate inter-subject comparisons in this second phase, these 3 musicians were asked to perform an identical musical excerpt at 3 different speeds, which were based on the performance of another player from the first phase, for a total of 12 versions of the excerpt (4 players, each performing the excerpt at 3 different tempos). In both phases, the instrument used was a marimba, which is a type of xylophone (Figure 2a), so that the size of the bars would be comparable to the instruments used by some of the performers from Cameroon.

For the second case-study, I worked with 21 xylophonists from Central Cameroon (a region where I have conducted field research in ethnomusicology since 1999 (17, 18, 19)), who use two types of instruments: Tikar and Bedzan Pygmies play on banana-tree trunk xylophones (c and 2d), standing side by side in pairs, striking the ends of large bars loosely placed on banana-tree trunks, while Eton musicians play individually on multiple-resonator xylophones (Figure 2b), where the bars are attached to the frame and struck in the centre, organized in small ensembles ranging from 3 to 7 instruments. In order to preserve the performing conditions as close as possible to the normal conditions of performance, the measurements were realized in context (i.e. during live performances), in the villages, in outdoor conditions. The repertoire was based on the main pieces known by all of the instrumentalists within each village, and among different villages of the same ethnic group, to facilitate comparison. The identification of the pieces to be performed was based on interviews with the members of the different communities (musicians, elders), cross-referenced between different groups and villages, as well as on recordings and transcriptions, following methods described in (20) and (21). However, although the original goal of this study was to compare Western and African xylophone players, the measurement conditions in Cameroon (bright sun light, position of the instrument requiring downward gazes with the eyelids being half-closed) led to a dataset that was not consistent enough after post-processing, reducing the data to 5 musicians only, coming from 3 different ethnic groups. As a result, this second case-study reports observations based on individual players rather than on inter-subject comparisons.

### Participants

All participants volunteered to take part in the study and were remunerated for their time.

In Canada, the percussionists were recruited through flyers and emails among the university percussion community and alumni. There were 1 female and 10 male participants, between the ages of 18-28 (average 23.4), with a minimum of 9 years of practice (average 13.7), all studying or having previously studied in Montreal. Three were left-handed. Nine university students in percussion performance (from Bachelor to Doctoral levels) took part in the first phase of the study. Three percussionists, active professionally as orchestral musicians and having all earned a master’s degree in percussion performance, took part in the second phase of the study.

In Cameroon, the musicians were selected by their respective communities, from which they were reputed to be experts. All were male, according to the vernacular traditions, and age range was between 22-60 years (average 38). All were right-handed. In the Eton area, two different xylophone ensembles were studied. In each ensemble, one soloist playing the xylophone in the highest range led the group, accompanied by one or two players performing on medium range instruments, and another player on the bass instrument (the number of bars on the xylophones decreases with the tessitura). In both cases, a drummer and a shaker player completed the ensemble, but only the xylophonists’ gazes were measured. Among the Tikar, I collaborated with a total of 6 pairs of xylophone players (two players play side by side on one xylophone) located in 5 different villages from the southern part of the Tikar area. One pair of players was measured among the Bedzan Pygmies, who represent a very small community (approximately 400 people in total) with a very vibrant and original musical culture. Bedzan and Tikar have close social relationships and use most of the same musical instruments (17).

### Technical Set-up

Measurements were realized with a mobile eye-tracker system (ASL MEXG) running at 60Hz. Performers wore a Mobile Eye XG Spectacle Mounted Unit consisting of an eye camera, scene camera, adjustable hot mirror, and a microphone, mounted on safety glasses, and secured by a headband to minimize displacement during performance. The glasses were connected by a cable to a Display/Recording Unit powered by battery that could be either worn at the belt by the performer or held by the researcher standing a few feet away (all performers chose the second option). In addition to the mobile-eye device, every session was recorded with a video camera (Panasonic HC-V700) placed in front of the player(s), connected to an external stereo microphone (AudioTechnica 2022). Eye-tracking data was captured with ASL EyeVision software (version 6.0.8.1) and analyzed with the ASL Results Plus GazeMap module (v. 1.8.3.15).

### Musical Material and Procedure

In Canada, the testing procedure involved a warm-up phase, the explanation of the procedure and system (including a demonstration on myself), the recording of the agreement to take part in the experiment, and the recording of different musical tasks, after a period of time devoted to the adaptation to the device, and calibration. An entire recording session did not last more than 60 minutes.

**Figure 3.**
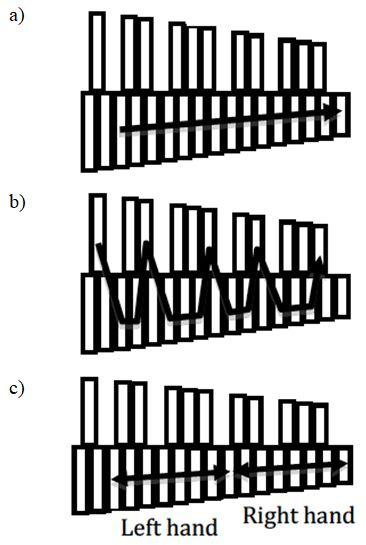
Top-view of the trajectory of the hands/mallets of the percussionist for different musical tasks: a) hands playing in strict alternation in a straight line (C-major scale); b) hands playing in strict alternation along a jagged-line to reach altered notes (B-flat major scale); c) hands playing in strict alternation but going in opposite directions back and forth from a central note (C) along the C-Major scale.)Top-view of the trajectory of the hands/mallets of the percussionist for different musical tasks: a) hands playing in strict alternation in a straight line (C-major scale); b) hands playing in strict alternation along a jagged-line to reach altered notes (B-flat major scale); c) hands playing in strict alternation but going in opposite directions back and forth from a central note (C) along the C-Major scale.

A series of 10 tasks was performed, typical of exercises practiced during years of training and used for warm-up before a performance. They represented different types of movements (Figure 3): 1) hands playing in strict alternation and moving up and down the register of the instrument in a straight line (C-major scale; Figure 3a); 2) hands playing in strict alternation and moving up and down the register of the instrument along a jagged-line to reach altered notes (B-flat major scale; Figure 3b); 3) hands playing simultaneously in parallel motion, a third or an octave apart (both hands striking simultaneously on an interval of a third – 1 bar between the two bars that are struck – or in octaves – 6 bars in between; Figure 3a or 3b, depending on the scale used, C-major or B-flat major); 4) hands playing in strict alternation but going in opposite directions back and forth from a central note (C) along the C-Major scale (Figure 3c).

The last task consisted of the performance of a musical excerpt well-known to each performer. Each task was performed at 3 different speeds: slow, medium and fast, the exact tempo varying with the level of each performer.

**Figure 4.**
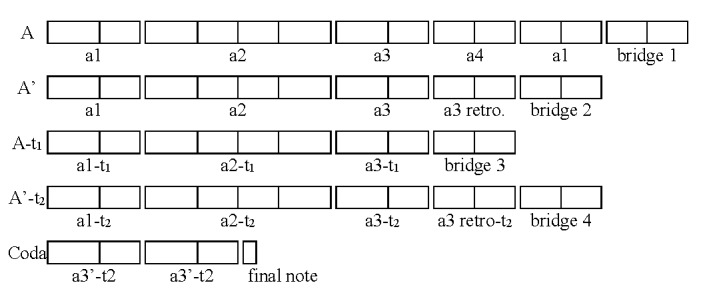
Musical structure of the excerpt from the xylophone part of ‘Porgy and Bess’, by G. Gershwin. Each rectangle represents one beat (four sixteenth notes); identical letters and indexes (e.g. a1) denote the repetition of the same musical material; ‘bridge’ indicates a musical section that leads to the following section with different musical material; ‘retro’ means ‘retrograde’ and indicates an inversion of the original pattern; ‘t’ denotes a transposition of the musical material, i.e. a translation of the musical pattern a few bars to the left or to the right on the keyboard of the xylophone; a3’ is a variation on a3, mainly a reorganization of the 3 sub-patterns that constitute this segment

During the second phase of measurements, we asked the 3 players to play a well-known excerpt from the orchestral repertoire, ‘Porgy and Bess’ by G. Gershwin, which presents several interesting characteristics:

1) it consists of a constant flow of 209 notes (sixteenth notes), played at a fast tempo, where the absence of longer notes or rests continuously solicits the attention of the performer;

2) the musical structure (Figure 4) is based on small segments of 2 or 4 beats that can be grouped in 4 larger sections (A, A’, A-Transposed, A’-Transposed, Coda), each segment being made of patterns of 2 or 3 notes that are presented in various combinations: the end result is a melody that, for the performer, seems to be almost always the same, but presents constant variations. Some patterns come back at several points in the piece, identical or transposed (see for example segment a1, played thrice in its original form, and twice in each of its transpositions);

3) the musical content requires performers to regularly perform two strokes in a row with the same hand, combined with accentuation, which is one of the difficulties of the piece, as it requires faster motion between two consecutive strokes for the same hand, and greater control from the performer. Each musician chooses to perform these double-strokes at different points in the excerpt, choosing from many possible sticking patterns.

The level of difficulty of this musical excerpt requires players to spend a significant amount of time practicing this relatively short piece, in order to foster consistency in their playing.

In Cameroon, every session started with a general warm-up of the ensemble, performing a few tunes from their repertoire. At this stage, they were often joined by the rest of community, dancing and singing along. The procedure and the functioning of the eye-mobile system were then explained to the performers, with a demonstration on myself, before proceeding to record their agreement in video. Performers were then equipped (one at a time, in turns) with the device and had time to play while wearing the eye-tracking glasses, and get comfortable before calibrating the system. The next phase involved the performance of 1 or 2 pieces chosen from the common repertoire, each player being equipped with the eye-mobile device in turns: for the ensemble of 4 players, this means that we recorded the same piece 4 consecutive times. Among the Eton, we completed this phase by asking the soloists to perform 3 more musical tasks that were present in their performance style, as soloists of the ensembles and main improvisers: conjunct-degree scale, parallel thirds and parallel octaves, played at a speed of their choice.

### Analytical Method

**Figure 5.**
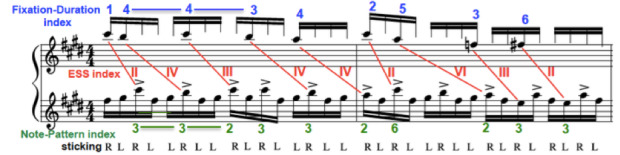
Example of a transcription of the excerpt of ‘Porgy and Bess’ (Player #2, 132 beats-per-minute) including: bottom musical staff, musical excerpt performed by the player; top staff, position of the gaze; in blue, the Duration-Fixation index; in red, the Eye-Stroke Span (ESS) index; in green, the Note-Pattern index; in black, the Sticking (R = right hand, L = left hand).

Data analysis was based on the videos that combine the scene-view, captured by the scene camera mounted on the glasses, with the point of the gaze on the scene, captured by the eye camera, after calibration and selection of the appropriate thresholds for each recording (corneal reflection spot recognition and pupil recognition). In this study, the absence of a musical score implies that musicians looked at their keyboard, or at their environment (other musicians, audience, etc.). In the first case-study (Canada), musicians looked exclusively at their keyboard during performance, which led to the realization of transcriptions that display two superimposed musical staves: one staff corresponding to the music that is performed, while the other presents the ‘musical score of the gaze’ (Figure 5).

Transcriptions were done by watching the videos at a reduced speed (6 times slower than the normal speed, i.e. 10fps instead of 60fps), every shift of the gaze being monitored frame by frame to observe accurately the timing of the shift in regard to the moment of the impact of the xylophone strokes. The transcription of the gaze trajectory according to a musical score implies that the duration of gaze fixation is indicated in terms of musical durations rather than in milliseconds, providing a note index that corresponds to the number of notes (or strokes in our case) during which the gaze remains fixated upon the same point on the keyboard; Furneaux and Land (10) describe it as a ‘note-unit performance index’, p. 2437. For both case-studies, the note index is based on the smallest subdivision of the beat, also called minimal operational value (the ‘smallest relevant duration obtained after subdivision’ ((15), p. 231)). In the first case-study, all the notes have the same duration and correspond to this minimal operational value, both in the scale-exercises and in the excerpt of ‘Porgy and Bess’, which made the use of the note index efficient and easy to apply. Note index was also easy to apply in the second case-study (Cameroon), where music is based on regular subdivisions of the beats. Measuring time based on a note index has the advantage of presenting data from the perspective of a performer, who constantly refers to musical durations during performance; it also facilitates comparisons between multiple performances of the same musical material, executed at different speeds.

From the transcription, we can extract several types of information:

- The total number of gaze fixations.

- The Fixation Duration, which corresponds to the time during which the gaze remains focused on the same position on the keyboard before shifting to the next fixation; Fixation-Duration may be expressed with a note index or in milliseconds.

- The Eye-Stroke Span (ESS), also measured with a note index, that corresponds to the duration of the anticipation of the gaze before a bar is struck. The eye-stroke span builds upon the notion of both eye-hand span and awareness span, which do not apply here because of the absence of a reference to a physical score: the keyboard of the instrument is indeed the only visual reference for the performer, who, while striking one bar, simultaneously looks ahead to the next bar (or set of bars) to be hit.

- The Note-Pattern index, which indicates the number of musical notes that are grouped together and performed in a chunk, before the gaze switches to the first note of the next chunk. The beginning of each note-pattern is determined by the note that is anticipated by the gaze, as identified with the eye-stroke span.

Fixation Duration, Eye-Stroke-Span and Note-Pattern are interrelated, yet independent from each other: for example, a Fixation Duration of 4 notes does not mean that the corresponding Note-Pattern will be of 4 notes (beginning of Figure 5); ESS duration also varies from one fixation to the next.

Finally, it should be noted that using musical notation to represent gaze-point in transcription reduces the accuracy of the data, due to the relatively large surface of a bar. In fact, the gaze is often positioned on the edge of a bar, or in the space between two bars, even on the very next bar as is the case at the end of Figure 5: the gaze is fixed on the very edge of the F-natural, close to the E, which is why the ESS is linked to the next E in the lower staff. In such cases, comments need to be added to the transcription to keep track of the decisions made during the analytical phase.

## Results - Case-Study 1

The results of the first case-study, dedicated to Western players, are based on the analysis of three of the musical tasks: 1) a C-Major scale, played in strict alternation of the hands over two-octaves, starting and finishing on the central C; 2) an exercise based on the same scale, where the hands move in opposite directions, going in progressively larger sweeping motions from the central C to the next octave (Figure 3c and Figure 9); 3) an excerpt of ‘Porgy and Bess’ (Gershwin).

**Figure 6.**
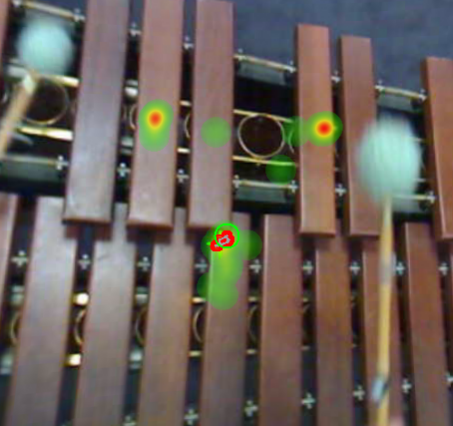
Heatmap of a short excerpt of a musical piece where the xylophonist (Player #2) plays on both natural and accidental keys. The focal point differs between natural keys (the musician’s gaze aims at the section where the accidental keys are superposed to the natural keys), and accidental keys (musician’s gaze aims at the middle of the bar, at the point of impact).bar, at the point of impact).

The first general finding is related to the position of the gaze on the keyboard: when playing on the natural keys, percussionists all fixed their gaze on the portion of the keyboard close to the area where both natural and accidentals (‘white’ and ‘black’ keys) are slightly superimposed, whereas they usually focused on the middle of the accidental keys when they played on those bars. Additionally, when playing several consecutive strokes combining natural and accidental keys within one gaze fixation, they often fixed a position on the bar that was close to the very corner of a bar, and located at the centre of an area covered by two or more consecutive strokes (Figure 6).

Main findings reveal a correlation between performance speed and a) the number of fixations, b) changes in lateral gaze movements for certain players. The comparison of 4 players for the musical excerpt of ‘Porgy and Bess’ gives insights on: a) the high reproducibility of gaze patterns for each individual across several performances, these patterns being nonetheless extremely individualized, and b) the relationship between Fixation-Duration, Eye-Stroke-Span and Note-Pattern.

The first task consisted of the execution of a C-Major scale, played in strict alternation, going up over two-octaves before returning to the original note, with 4 notes per beat for a total of 29 notes. All performers were asked to play at slow, medium and fast tempos; some also performed at an “extra-fast” tempo. Table 1 displays the individual speeds chosen by each performer for each speed category, along with the total number of eye fixations; there was no significant difference in the number of eye fixations depending on the trajectory of the musical scale (left to right for the first part of the exercise when pitches go up, right to left for the second part), t(31) = 1.57, *p*=0.12, (two-tailed paired Student’s t-test).

**Table 1.** Number of eye fixations for each performer for the C-Major scale exercise, according to different speeds (in beats per minute, or bpm).

	Tempo (bpm)						Number of fixations (entire scale: up/down)				
Player		Slow	Medium	Fast	Extra-fast		Slow	Medium	Fast	Extra-fast
1		48	82	146	163		26	21	13	11
2		75	114	150			19	14	12	
3		40	60	110	188		27	24	15	11
4		42	88	166			29	16	12	
5		68	104	126			21	15	12	
6		82	110	150			18	17	11	
7		80	132	187			21	12	10	
8		81	126	182			17	11	8	
9		48	92	138	166		24	17	12	7
10		85	120	160			20	17	11	
11		59	92	143			27	22	13	
Average		*64*	*102*	*151*	*172*		*23*	*17*	*12*	*10*

**Figure 7.**
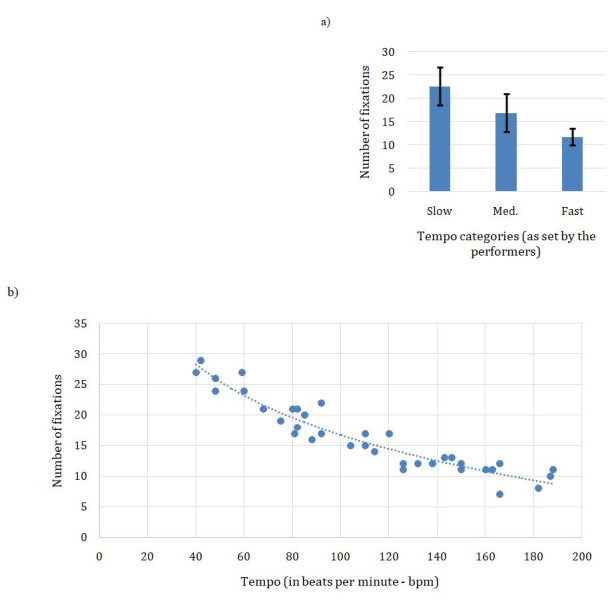
C-Major scale over two octaves in strict alternation of the hands. a) Mean number of eye fixations (y-axis) in relation to tempo conditions (x-axis: Slow, Medium, Fast) according to performers’ preferences; error bars represent the standard deviation. b) Number of eye fixations (y-axis) in relation to performance tempo across all trials, in beats per minute (x-axis).

While the interpretation of ‘slow’, ‘medium’ and ‘fast’ varies significantly from one performer to the next, it is interesting to note that the number of eye fixations decreases with the augmentation of speed for all performers (Figure 7a). At a slow tempo, the xylophonists take the time to fix their gaze on most of the bars (up to 29 fixations for a total of 29 strokes). At a medium tempo, the decrease in the number of fixations varies quite significantly from one performer to the next, and at a fast tempo, half of the performers have decreased their number of fixations by half compared to the slow tempo. The extra-fast category sees further decreases in the number of fixations, and it should be noted that players start to lose accuracy.

A one-way ANOVA revealed a significant effect of tempo condition (Slow, Medium, Fast) on the number of fixations (*F*(30,2)=26.73, *p*<0.0001) as shown in Figure 7a. Post-hoc tests revealed significant differences across all three conditions (*p*<0.005). The ‘Extra-fast’ condition was not taken into consideration in this comparison since only 3 participants added this fourth category.

These results indicate that fixations are correlated to speed, typically going from a fixation for every note or almost every note (every xylophone bar) at the slowest tempo, to almost 3 notes at the fastest tempo. At the slowest tempo (42bpm), player #4 realizes 29 fixations, the duration of each note being 375ms, while at the opposite end of the spectrum in the ‘fast’ category (187bpm), each note is 80ms long and player #7 uses 10 fixations to perform the entire exercise, for a Fixation-Duration mean of 232ms; while the duration of each note is more than 4 times shorter in the second case, the number of fixations is not reduced proportionally. Figure 7b reveals a strong correlation between the augmentation of performance speed and the reduction in the number of fixations (r(34) = 0.907, *p <0.01*) which does not linearly scale but rather follows a logarithmic function.

Comparing the ratios between ‘slow’, ‘medium’ and ‘fast’ categories for performance speed and their corresponding inversed ratios for gaze fixations (Table 2) helps to underline that ratios vary considerably from one player to the next (see for example players #4 and #10 in the comparison between ‘slow’ and ‘fast’ speeds). Moreover, there are few exceptions where the ratio is similar (player #8 for the ‘slow’/‘medium’ comparison), or inverted (players #7, ‘slow’/‘medium’; players #5, #6, #10 and #11 for the ‘medium’/‘fast’ comparison).

**Table 2.** Comparison of the ratios between different performance speeds (Slow, Medium and Fast) and the ratio for the number of fixations, for each performer for the C-Major scale exercise.

	Ratio Medium/Slow			Ratio Fast/Medium			Ratio Fast/Slow	
Player	Performance Speed	Number of Fixations		Performance Speed	Number of Fixations		Performance Speed	Number of Fixations
1	1.71	1.24		1.78	1.62		3.04	2.00
2	1.52	1.36		1.32	1.17		2.00	1.58
3	1.50	1.13		1.83	1.60		2.75	1.80
4	2.10	1.81		1.89	1.33		3.95	2.42
5	1.53	1.40		1.21	1.25		1.85	1.75
6	1.34	1.06		1.36	1.55		1.83	1.64
7	1.65	1.75		1.42	1.20		2.34	2.10
8	1.56	1.55		1.44	1.38		2.25	2.13
9	1.92	1.41		1.50	1.42		2.88	2.00
10	1.41	1.18		1.33	1.55		1.88	1.82
11	1.56	1.23		1.55	1.69		2.42	2.08
*Average*	*1.62*	*1.37*		*1.51*	*1.43*		*2.47*	*1.94*

**Figure 8.**
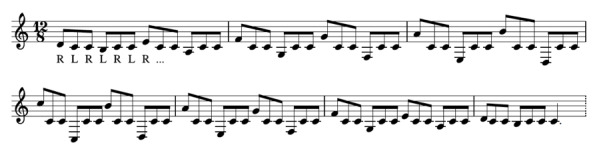
Musical representation of the exercise where Left Hand (L) and Right Hand (R) move progressively away from the central note (C), but constantly having to come back to it

The next results refer to an exercise based on the same scale (C-Major), where the hands move in opposite directions, making progressively larger sweeping motions from the central C to the next octave (Figure 3c and Figure 8). Performance speed was chosen by the players according to the same categories (Slow, Medium and Fast, as well as Extra-fast for a few players who performed another run).

**Figure 9.**
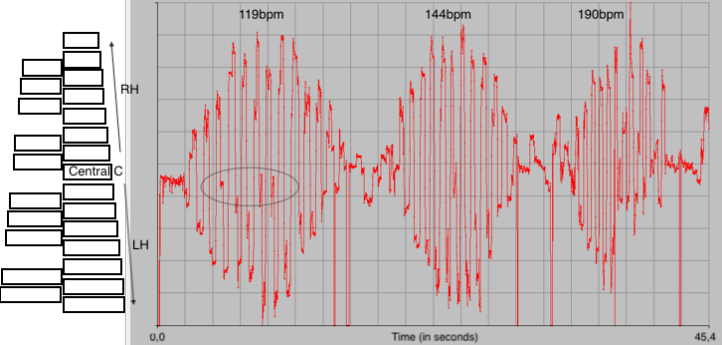
Representation of horizontal gaze coordinates for the contrary motion task based on a C-Major scale, performed at 3 different speeds (from slow to fast) by Player #2. The upper part of the schema represents the high register, the highest point being the highest note (towards the right of the performer), the bottom part represents the low register (towards the left of the performer) – the drawing on the left represents a top-view of the keyboard. At the slowest speed, 119bpm (left), the musician has enough time to fix on the central note (cf. circled section). At medium speed (144bpm), there is only one fixation on the centre of the keyboard, while the last iteration of the task (on the right, 190bpm) leads to a loss of control of gaze accuracy – which, in this case, takes place on the non-dominant hand side of the player, before fixing only on the central note of the exercise, C.

For this exercise, players tend to cast their gaze ahead by 3 notes (ESS note index of 3): when the right hand reaches a high note, the gaze shifts immediately to the next lowest note to be played by the left hand, skipping the two central notes (C) that are played in between; however, there is an influence of performance speed on the eye-movements of the players. At slow speeds, xylophonists have enough time to look at every bar and anticipate not only the notes that are at the extreme end of the range that they need to cover, but they also focus on the central note of the exercise (C). When speed increases, they skip this intermediate step to jump from one extreme to the next, and when they reach the limits of their skills, their gaze tends to lose focus and synchronicity with the bars to hit, leading to pitch errors (Figure 9) or they stop moving their eye laterally to focus only on the central note of the range (C).

As was the case for the first set of results, there are numerous discrepancies between performers who use different strategies: while some of them try to anticipate all of the striking positions at all speeds, i.e. extremes and centre of the range (Table 3, Player #6), some stop switching their gaze from left to right when they reach a certain speed, fixing their gaze only on the central note C (Players #1, #4, #10 and #11). Player #8 begins applying this technique at the slowest speed. In general, the gaze-stop on the central note is associated with slower speeds, while the unique focus on the central note is associated with higher speeds, but the gaze-stop technique reappears at higher speeds when performers start to make some errors on this note.

**Table 3.** Characterization of gaze-fixations strategies for each player according to performance speed. ‘Gaze-stop on central C’ corresponds to a fixation on the central note of the keyboard, which is repeated twice in between extreme pitches. ‘Focus on central C only’ refers to players who only fix their gaze on the central note of the keyboard, while playing all of the exercise. ‘Y’ indicates that performers use this technique, ‘N’ that they do not have recourse to it. ‘(Y)’ indicates partial use through the entire exercise. ‘N’ in both categories corresponds to gaze-shifts from one extreme to the other, the gaze never fixing the central note except for the very beginning and end of the exercise.

	Tempo (bpm)						Gaze-stop on central C						Focus on central C only				
Player		Slow	Med.	Fast	Extra-fast		Slow	Med.	Fast	Extra-fast		Slow	Med.	Fast	Extra-fast
1		120	174	234			Y	N	N			N	(Y)	Y	
2		119	144	190			Y	N	(Y)			N	N	N	
3		73	104	162	225		Y	Y	Y	N		N	N	N	N
4		104	220	254			Y	N	(Y)			N	Y	(Y)	
5		112	153	177			(Y)	N	N			N	N	N	
6		122	140	178			Y	Y	(Y)			N	N	N	
7		104	170	240			Y	N	N			N	N	N	
8		117	164	208			N	N	N			(Y)	Y	Y	
9		108	156	194	211		Y	Y	N	N		N	N	N	N
10		136	172	216			(Y)	N	N			N	N	(Y)	
11		150	202	242			Y	N	N			N	N	(Y)	

This second set of results indicates that performance speed has an impact on the way performers fix the keyboard with their gaze, in this instance on lateral shifts. Xylophonists use different techniques with their gaze to combine accuracy and performance speed, in turn or in partial combination throughout the exercise: fixing on all of the striking spots, shifting between extremes, or focusing only on the central note of the range.

The third set of results is based on the analysis of the musical excerpt of the xylophone part from ‘Porgy and Bess’. Based on the performance of the first player (Player #2), three other xylophonists recorded the excerpt at the same respective speeds: 127bpm, 132bpm and 144bpm, all being within the range of an actual performance tempo with an orchestra. Data loss occurred once for each player, at different tempi: 6 notes for Player #1 at 127bpm (including one blink); 4 notes at 144 for Player#2; 6 notes at 144bpm for Player #3; 2 notes at 132bpm for Player #4 (blink). The data was either taken out of the results, or compensated by checking the eye-camera video, to detect any displacement or change in the gaze: if the eye and the head were both immobile, as could be verified respectively with the eye-camera and the scene-camera, it was assumed that the fixation was continuing through the data loss; else the data was discarded during the corresponding frames.

**Figure 10.**
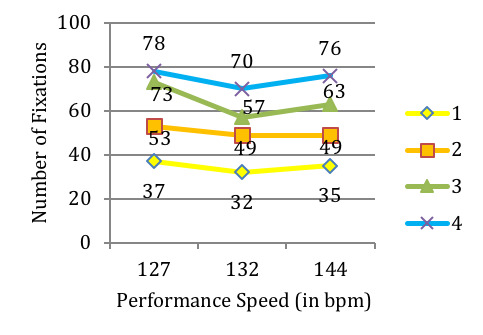
Comparison of the total number of fixations for the musical excerpt of ‘Porgy and Bess’ for 4 players across 3 performance speeds: 127, 132 and 144 beats per minute (bpm). Players are identified by their number (#1 to #4).

The number of fixations over the entire musical excerpt varies greatly from one player to the next (Figure 10): Player #1 uses less than half the fixations than Player #4. All players use more fixations at the slowest speed, however the number of fixations is the lowest at the intermediate speed for three of them. Player #3 displays the largest amount of variability in the number of fixations across the three versions (21.9%).

**Figure 11.**
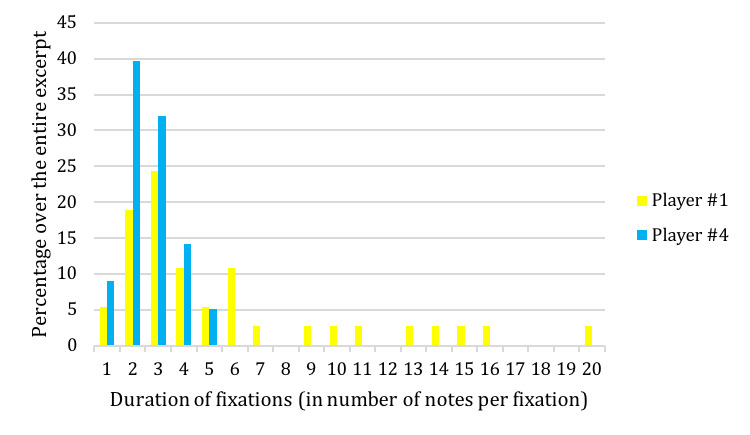
Comparison of Fixation-Duration (in % of total number of fixations over the total excerpt) for Player #1 and #4 at 127bpm for the musical excerpt of ‘Porgy and Bess’.

The variability in the total number of fixations depends on the duration of these fixations: less fixations in total implies longer single fixations, although the Fixation-Duration observed for Player #1 are not systematically twice as long as the Fixation-Duration of Player #4. The comparison of the Fixation-Duration for these players shows that Player #4 relies solely on short Fixation-Duration of 1 to 5 notes, while Player #1 uses fixations that can last up to 20 notes, i.e. the equivalent of 5 beats or 2.36 seconds (Figure 11). The longest Fixation-Duration was found in the performance at 132bpm of Player #1, with fixations lasting 23 and 24 notes (2.72 seconds) in musical sections A (a1 + a2) and A’-t2 (a1-t2 + a2-t2) (Figure 4).

**Figure 12.**
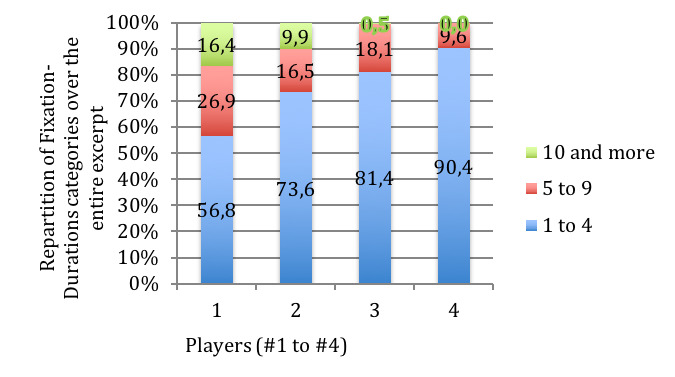
Comparison of Fixation-Duration (in % of total number of fixations over the total excerpt, y-axis) for all players across all tempos for the musical excerpt of ‘Porgy and Bess’. Players are numbered 1 to 4 (x-axis), and Fixation-Durations are grouped in 3 categories: 1 to 4 notes, 5 to 9, 10 and more.

When comparing individual players across versions, it appears that longer Fixation-Durations take place in the same musical segments, which is not true when realizing an inter-subject comparison. To facilitate comparison between versions and subjects, Fixation-Durations were grouped into 3 categories chosen to reflect recent findings by Cara about the limits of musical information processing of 7 +/- 2 notes (13), that confirm Sloboda’s findings (8): 1 to 4 notes, 5 to 9 notes, 10 and more. These categories create the opportunity to obtain a profile for each player, depending on their tendency to use shorter or longer Fixation-Durations (Figure 12). The influence of speed on the variation of Fixation-Duration for each category remains very individualized, each player displaying different variations from one speed to another.

As mentioned earlier, Fixation-Duration, ESS and Note-Pattern are interrelated, but not identical.

**Figure 13.**
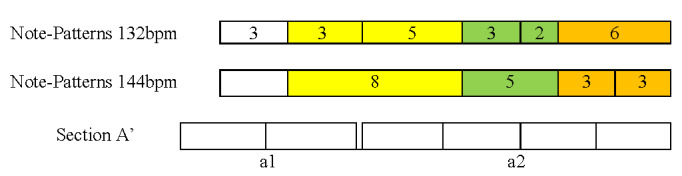
Example of the amalgamation of note-patterns from one version (132bpm) to another (144bpm), Player #2, beginning of the A’ section (segments a1 and a2) of the musical excerpt of ‘Porgy and Bess’.

Such is the case for the relationship between Fixation-Duration and Note-Pattern: if a fixation is 5 notes long, it does not mean that the corresponding Note-Pattern will have the same duration (see Figure 5, for example); however, there is a strong link between both, and the longest Note-Patterns (24 and 25 notes long) are found in performances by players who have the longest Fixation-Durations. An interesting characteristic of Note-Patterns is that their segmentation remains relatively stable across versions for each of the players, who tend to amalgamate small chunks in longer patterns. The comparison of the versions at 132bm and 144bpm at the beginning of section A’ for Player #2 is a good example (Figure 13): from one version to the other, Player #2 amalgamates two chunks that are 3-note and 5-note long into a single chunk that is 8-note long (highlighted in yellow in Figure 13), ), proceeding in the same manner for the following chunks (highlighted in green). This process is not systematic, as can be seen with the last note-pattern in Figure 13, where a 6-note long Note-Pattern is divided in 2 sub-groups (in orange).

**Figure 14.**
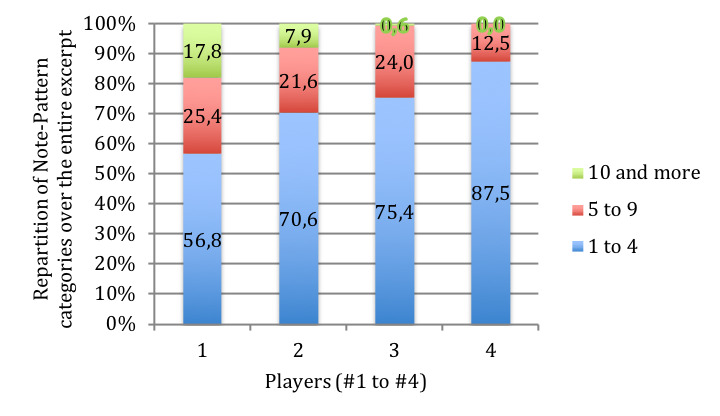
Comparison of Note-Pattern categories (in % of total number of fixations over the total excerpt) for all players across all tempos for the musical excerpt of ‘Porgy and Bess’. Players are numbered 1 to 4 (x-axis), and Note-Patterns are grouped in 3 categories: 1 to 4 notes, 5 to 9, 10 and more.

The categorization in 3 classes (1 to 4 notes, 5 to 9, 10 and more) has also been applied to Note-Patterns, to compare versions and players. For the intra-subject comparison, the influence of speed on the variation of Note-Pattern durations remains very individualized, each player favorizing different variations from one speed to another. The inter-subject comparison presents a profile quite similar to the Fixation-Duration, but the category 5 to 9 notes is larger for Note-Patterns than for Fixation-Duration for all players except for player #1 (Figure 14).

**Figure 15.**
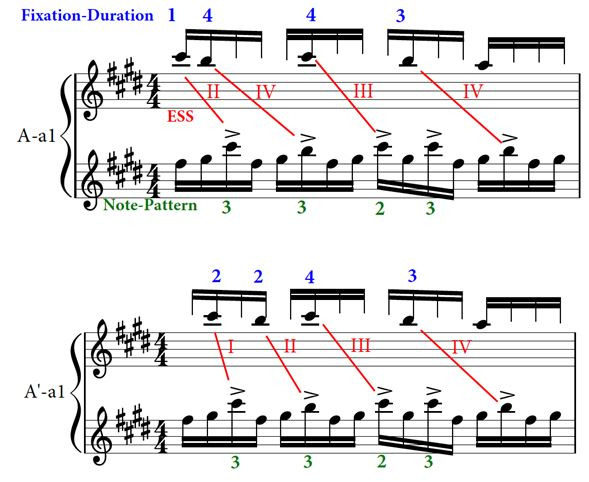
Comparison of the relationship between Fixation-Duration, Eye-Stroke Span and Note-Pattern in two identical musical passages of the musical excerpt of ‘Porgy and Bess’ for Player #2 (127bpm). The Note-Pattern segmentation remains identical while the Fixation-Duration and ESS vary.

The possibility for a musician to perform identical musical passages with the same Note-Pattern segmentation, but with different Fixation-Duration, is based on the flexibility of the Eye-Stroke Span: such is the case, for example, for Player #2 in the musical segment a1 in sections A and A’, at 132bpm (Figure 15).

**Figure 16.**
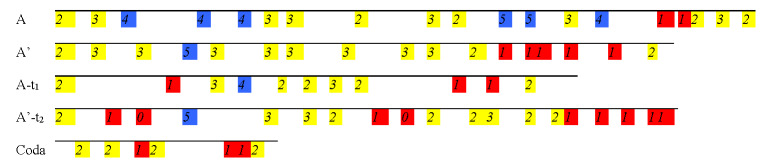
Overview of the anticipation measured with the ESS for Player #3 (127bpm). ESS categories are color-coded with red for an ESS of 0 to 1; yellow for 2 to 3; blue for 4 and more.

Duration of ESS vary from 0 to 6 notes across all versions, 0 indicating a moment when the player looks at the bar at the moment of the strike, which happens rarely. The majority of ESS have a duration of 2 or 3 notes. ESS durations can be grouped in 3 categories: 0 to 1 note, which indicates minimal gaze anticipation; 2 to 3 notes, corresponding to the majority of cases and to the strategies observed in the scale exercises; 4 and more notes, indicating a gaze anticipation of at least one beat. By color-coding these 3 categories, it is possible to produce a sort of ‘map of anticipation’, where red corresponds to minimal anticipation and blue maximal anticipation (Figure 16), that helps to proceed to intra-subject comparison. Such comparisons show that players tend to exhibit shorter or longer anticipations in the same musical passages, beyond variations in ESS.

**Figure 17.**
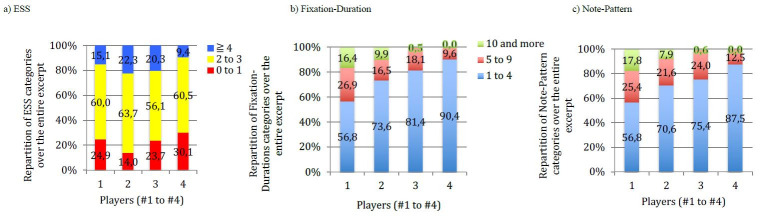
a) Comparison of ESS categories (in % of total ESS over the total excerpt) for all players across all tempos for the musical excerpt of ‘Porgy and Bess’. Players are numbered 1 to 4 (x-axis), and ESS are grouped in 3 categories: 0 to 1 note; 2 to 3; 4 and more. b) and c) correspond to Figures 12 (Fixation-Duration) and 14 (Note-Pattern), reported here to facilitate comparisons between players

A comparison between all players across all versions reveals that all performers favour an ESS of 2 or 3 notes, but that the ESS seems to be more independent of the other variables, as demonstrated by the repartition of categories across players that differs from Fixation-Duration and Note-Patterns graphics (Figure 17).

## Results - Case-Study 2

The results of the second case-study are based on measurements and observations realized among 3 different ethnic groups in Cameroon. As aforementioned, data quality was uneven after post-processing and the results are based on a few individual players, rather than on inter-subject comparisons. However, even when the eye-tracking data were not exploitable, the image of the eye-camera provided precise information on the opening or closing of the eye, and the scene camera, combined with the other video camera placed in front of the player, reported additional information on the orientation of the head and of the gaze of the performer.

The main findings are linked to the time musicians spend looking at or away from their instrument, and to ESS. The main difference between xylophonists observed in Cameroon and in Canada is that Cameroonian players do not always look at their keyboard while playing. Two types of performance practices can be distinguished, based on the variety and the complexity of the music being performed. Among the Bedzan and Tikar, players use banana-trunk xylophones, where each person strikes a limited range of 4 to 6 notes. The xylophone bars are also quite large at the striking position (6-9 cm), and as the music is extremely repetitive with limited melodic variations, the necessity to look at the instrument to improve accuracy is less prevalent for those musicians. The same conditions apply to xylophonists who have an accompanying role in Eton music, though the musical material presents more variations (pieces include several sections that create more melodic and rhythmic variations; there is more interaction between players who react to one another’s variations; an accompanist might take short solos). The situation is somewhat different for the soloists whose role is to lead the Eton ensembles: their musical task is divided between sections in which they play repetitive patterns with few variations – often singing at the same time – and sections in which they improvise on their instruments, without singing. During the singing or steady pattern sections, they look at their accompanists (looking at their faces or at their hand motions) or at the audience, whereas during the solo sections, they look at their own xylophone.

Most players spend a significant amount of time looking away from their keyboard: in Bedzan and Tikar villages, musicians generally look away from their instrument 60 to 75% of the performance time. Their gaze is focused on singers or dancers surrounding them, or on their fellow xylophonists’ sticking patterns. Recordings in the Tikar village of Ngambé showed that when musicians were looking at their own sticks or bars, the time spent on their zone of playing ranged from 0.2 to 3 seconds, with the vast majority of time spent on this zone under the duration of a full musical cycle (around 2 seconds). It is also not rare for musicians to close their eyes during a performance, and one of the most experienced xylophonists among the Tikar was observed fully closing his eyes over the span of 20 consecutive seconds, which represents 10 iterations of the musical cycle.

**Figure 18.**
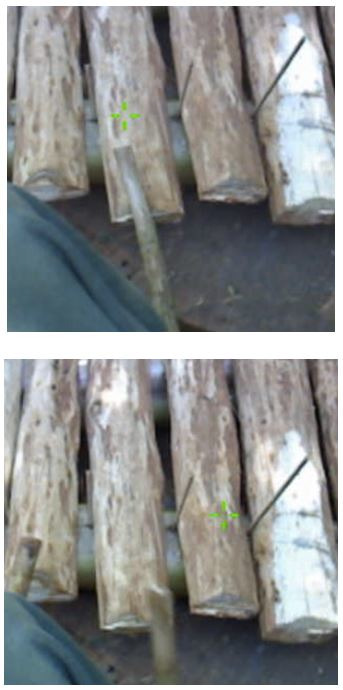
Two illustrations of the gaze-point on a banana-trunk xylophone played by Bedzan. On top, the point of gaze is positioned just beyond the extremity of the stick; on the bottom, the gaze is anticipating the next strike of the right hand by an ESS of 3 notes.

When they look at their keyboards, Bedzan and Tikar focus their gaze close to the point of impact (at the extremity of the bar) or just beyond the extremity of their stick or mallet (Figure 18). Eton players also focus close to the point of impact, which is in the middle of the bar for their xylophones.

**Figure 19.**
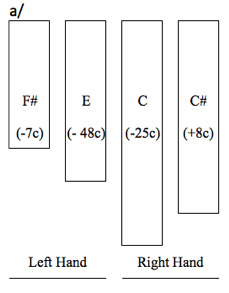
a) top-view representation of the 4 bars of the xylophone played by the Bedzan xylophonist, with their pitch and deviation in cents from equal temperament (a *cent* is a hundredth of a semi-tone); b) transcription of the basic pattern; c) transcription of a variation of the musical pattern (bottom line), with the musical score of the gaze superimposed (top line). Numbers 1 to 6 refer to the beats of the musical cycle.

Among Bedzan Pygmies, the performer playing in the high range executed a short cycle of 6 beats subdivided in 3 notes at 178bpm, using only 4 bars (minimal operational values are 112ms long), the cycle being repeated with small variations. Each hand struck one of the two central bars to execute the basic pattern, while the two bars on the outside were used to add variations, generally on the sixth beat of the cycle. Eye movements were limited, focusing mainly on the bar played by the right hand during the basic pattern, leading to Fixation-Duration lasting most of the cycle, with slight repositioning on the bar every beat or second beat. The main gaze shifts occurred one or two beats before the variation (ESS of 3 or 6), the Fixation-Duration on the new note lasting for an entire beat (Figure 19), as well as the Note-Pattern. Occasional gaze shifts also took place towards the bar played by the left hand in the basic pattern, with an ESS of 2 or 3 notes, and for a Fixation-Duration of 2 to 3 notes also.

The solo xylophones in Eton ensembles count up to 20 bars, tuned in a heptatonic scale. In solo sections, the xylophonist executes melodic and rhythmic variations that include techniques such as passages in parallel thirds or octaves (simultaneously playing bars that are respectively set apart by one bar, or by six bars), or rolls on ascending or descending scales, which consist of a fast series of repeated strikes on each bar before moving to the next. These techniques could be extracted from their original context and executed separately to record specific eye-movements. For parallel thirds, the performer repeated each double-stroke once before moving to the next double-stroke, playing two double-strokes per beat at 164bpm, in a general ascending motion from the lowest bar to the highest bar (for Eton players, this means playing from the right to the left, the lowest bars being positioned to the right of the performer). The player consistently looked at the usual point of impact of the mallet with the bar, i.e. at the centre of the bar. For the first part of the exercise (6 beats), the performer had to hit the bars positioned on his right side and focused his gaze on the bar that separated his mallets. A shift occurred around the 7th beat, while he was playing on the bars located in front of his body, and his gaze shifted towards the bar that was struck by the left hand (leading the motion towards the upper register). Gaze shifts took place after the repetition of the second double-stroke, the gaze shifting to the next fixation just before the beginning of the next set of double-strokes, for an ESS of 0.5. Gaze shifts lasted 120 to 150ms.

For roll sequences on continuous scales, the performer repeated each note 3 or 4 times before changing note, playing 14 to 15 strokes per second. The gaze was always focused on the first or second bar to the left or to the right of the bar that was played, depending on the global motion (towards the upper or the lower register). ESS was of 3 or 4 notes. Saccades were 33 to 50ms long, a duration comparable to those reported in Lehman and Kopiez (22), and they usually took place right after the last stroke of a group of repeated notes on the same bar.

It should be noted that the data corresponding to the portion of the keyboard located in front of the body of the player was lost.

## Discussion

### Looking at the Keyboard, Looking Away

In both countries (Canada and Cameroon), it was possible to observe where xylophonists look during a performance: an emphasis on interaction with their surroundings was found for African xylophonists, versus a more instrument-centered approach for Western performers. However, the nature of the music performed was extremely different. It is interesting to note that when the musical task required more focus from the Eton solo players, they were looking at their keyboard, similar to Western performers playing exercises and musical excerpts that require perfect accuracy. Musicians from Cameroon who were playing extremely repetitive musical tasks displayed a great freedom in the use of their gaze. This behaviour is also linked to cultural factors: it is considered normal to perform a musical instrument without having to look at it and Tikar in particular purposely look away from their keyboard (see, for example (23) for the relationship between musical posture and culture). In Western performance, some players also developed this attitude, as reported by Singer (24) about American xylophonist Red Norvo, who learned to play without looking at his hands to make eye contact with the audience, as advised by his agent. In Cameroon, the xylophonists also commented on the fact that they needed to be able to play at night, with poor light sources – for examples for festivals, wedding celebrations, etc., in both interior and exterior venues. According to several players, “a skilled performer should be able to play in the dark”: this claim might be true for players who execute near automated musical tasks but would need to be verified.

When musicians looked at their instrument, the localization of the performer’s gaze on the keyboard of the xylophone was assessed. All performers look at the point of impact of the mallet with the bar, or just beyond this point: for Western performers, they look close to the overlap between accidental and naturals when they play on natural keys, rather than on the centre of the keys for accidentals. Most musicians from Cameroon look at the point of impact, though among the Bedzan musicians, we observed gazes that were directed beyond the tip of the mallet.

### Eye/Hand Synchronization

Eye-hand synchronization involves the relationship of four elements: the number of fixations, Fixation-Duration, Eye-Stroke Span and Note-Pattern. The number of fixations, Fixation-Duration and Note-Pattern are closely interdependent: when a musician uses more fixations, the duration of the fixations (Fixation-Duration), and of the musical chunks that are anticipated within one gaze (Note-Patterns) will be shorter. These three elements may vary significantly between one player and another, as was found in the scale exercises and in the musical excerpts; the influence of speed on the changes from one tempo to another was also individualized. In conformity with previous findings (9), the number of fixations decreases when the performance speed is increasing in the context of musical scale exercises. However, in the musical context of ‘Porgy and Bess’, there is a decrease between the slow and medium tempos, but an increase between medium and fast tempos for 3 out of 4 players, the fourth player maintaining the same number of fixations. The total number of fixations remains lower at the fast tempo compared to the slowest tempo, for all players. This discrepancy between an exercise (scales) and the musical excerpt needs further investigation; it could be explained by the fact that the chosen musical excerpt requires a sequence of eye-movements that is constantly changing, both in terms of localization on the keyboard and directions, when compared to the scale exercises that involve progressive sequences, which might lead the players to augment the number of fixations when they are reaching their maximum speed in order to ensure the best possible performance accuracy.

The fact that Note-Pattern segmentation remains quite constant across different versions and tends to be identical within the same excerpt when the same musical material is repeated, reveals partially how performers build their mental system of reference. This observation correlates findings on the planning of long musical sequences that are ‘broken down into short segments that contain several events’ ((25), p. 28, referring to (26)). The question of how these blocks are built needs further investigation, but eye-tracking is certainly an excellent tool to provide insights in this domain.

The musical chunks delineated with Fixation-Duration and Note-Pattern are linked together by Eye-Stroke Span, which allows performers to anticipate their movement. ESS duration is mostly of 2 to 3 notes but may go as high as 6 notes for certain players, as was observed both with Western and African musicians. It can also be very short, i.e. 0 or 1 note: in such cases, the anticipation is either non-existent (0 note) or minimal (1 note). It is interesting to underline that by establishing ‘maps of anticipation’ and comparing them, the passages where a performer is able to make longer anticipations, or on the contrary always relies on short anticipation, provides another insight on how each player segments the piece to be performed, as well as potentially giving indications on passages that are more difficult to perform.

In the exercise involving opposite movements going back and forth from the central note C, some players who were not stopping their gaze on the central note during the performance looked at this note for 2 or 3 consecutive beats after making mistakes, in order to correct them. In this case, these extra fixations were used to correct errors that had already been performed, and the capability for a musician to change his pre-set flow of anticipation further demonstrates that flexibility is a characteristic of ESS.

It is also possible that the absence of anticipation denotes a phenomenon that could be equivalent to regressive fixations in music reading. In some of the transcriptions of ‘Porgy and Bess’, there were a few instances where a musician anticipated in the usual manner but looked again at the note to be played at the exact moment of the performance; in another instance, the musician anticipated on the wrong pitch, and took a second look just before playing the correct pitch. The fact that the player was stopping his constant anticipation to realize a second fixation for the same point in the music flow, is akin to a player looking back in the score to double-check an element in the score just before playing a note, thus avoiding a mistake. For Western percussionists playing from memory, as was the case for the excerpt of ‘Porgy and Bess’, the score has been internalized and the visual reference associated with the musical performance has been replaced by points of reference on the keyboard of the instrument. It would be interesting to know to what extent performers actually follow a mental visual representation of the score during their performance: such practice would further support the idea to compare these movements to regressive saccades in sight-reading (10).

During the current research, as well as in a previous study (unpublished), I asked the performers (40 percussionists total combining both studies) what they saw when they closed their eyes and imagined themselves performing on a xylophone or marimba. Two elements emerged: 1) a large majority of musicians were ego-centered (viewing the keyboard of the instrument from a personal perspective, akin to the view that is provided by the scene-camera of the eye-tracker (7)), while several were allo-centered (using a top-view, detached from their personal point of view), and very few "saw" themselves from the perspective of the audience, in a mirror-like manner (further questioning revealed that all of these particular performers had spent long hours of practice in front of a mirror to observe their technique); 2) in the first category (ego-centered), musicians reported different ways on how they "see" the instrument in their mental representation. For example, some include part of their mallets and hands in this mental image, while others see only the keyboard. The portion of the keyboard that is being visualised also varies from one player to the next, including all of the notes separating the two hands of the player, or integrating only a few notes at a time around each hand, these zones moving according to the positions of the hands over the keyboard. Finally, several players mentioned the xylophone bars to be played lighting up in succession, or visualizing trajectories on the keyboard that correspond to the sequence of notes to be played. These testimonies indicate that performers are able to describe the type of interaction that they have with their instrument, which complements the information that can be collected with eye-tracking technologies. They also provide some insight on the nature of the memory buffer mentioned by Kinsler and Carpenter (9), while raising questions that need further systematic investigation.

### Performance Threshold

All musicians have a superior limit beyond which they begin to play inaccurately. This limit is dependent on many factors, including the capacity to use their gaze properly to anticipate their actions. As the number of fixations decreases with speed, one potential threshold is linked to the minimum number of fixations that a musician needs to properly execute the task. In the C-Major scale exercise, for example, none of the players went below 8 fixations without making mistakes, and the maximal capacity of short-term memory is probably an important limiting factor here. An element supports this hypothesis: when tempo increases, the duration of musical notes decreases faster than the number of fixations; in other terms, there are more and more notes that are monitored within one fixation, until cognitive limits are reached.

In the C-Major exercise where the strokes move progressively away from the central note (Fig. 9), a similar effect was noted: at slow speed, performers have the time to rest their gaze on each bar they play, skipping the central note at higher speed or, on the contrary, focusing only on the central note, and no longer looking to the other notes. Players’ personal limits to execute extremely quick lateral movements of their gaze could explain why some are able to keep their gaze moving laterally at high speeds, while others opt for a strategy that consists of relying on their proprioceptive skills. Focusing on a single note might also reveal the capacity of some players to change their focus in order to enlarge their perceptual span. This could also explain why some xylophonists are able to execute long sequences of consecutive notes within one fixation.

### Limitations

The specificity of xylophone performance and the necessity to go in the field, in Cameroon, to perform some of the measurements were challenging and led to some limitations. One of the main problems, that I did not anticipate, was the fact that the glasses of the eye-tracker were not easily adaptable to the facial structures of several of the performers, both in Canada and Cameroon. Moreover, the nature of the task and the natural posture of the xylophonists create specific conditions where the eyes are often not fully open, masking part of the pupil, which interferes with the eye-tracking procedure. Although the calibration phase was based on looking at different positions on the xylophone, the musicians’ head position differed enough between the calibration and the actual performance to result in the loss of many recordings, especially in Cameroon. Musicians are indeed casting their gazes downwards, while maintaining their head relatively high. As a result, the eyelids are not always fully open, and the angle that allows the eye-tracking system to both project on the surface of the cornea three near-infra-red dots and to video-record the eye, is extremely narrow. In practice, even if the calibration phase is executed correctly, the musician’s gaze is directed in such a way that sufficient tracking of the eye-movement is not always possible. The light conditions were also not always optimal in Cameroon. All of these parameters could be better controlled in future research; however, it is necessary to find the balance between ideal eye-tracking conditions and ecological validity: what is gained on one side in the ecological validity of the task, may be lost on the stability of the experimental conditions. Finally, another limitation is linked to the technical capabilities of mounted eye-tracking systems: xylophone performance often implies quick gaze-shifts and large lateral saccades covering a large field of view, which also proves challenging for these systems.

The research presented in this paper was exploratory by nature, since it involved the use of a head-mounted eye-tracking system applied to an instrument that had not been previously studied in this manner, as well as a comparative approach involving different cultures. As a result, the original research question was quite open and the choice was made to give maximum freedom to the performers in their tasks: for example, whereas most studies control the tempo to facilitate comparison between musicians, the first phase in studio trials in Canada, as well as all of the recordings in Cameroon, were realized with the musicians performing according to their own speed categories. While this approach allows for observations that more closely resemble ‘natural’ performances (as much as a performer can feel perfectly at ease while being equipped with an eye-tracker), it implies a novel approach to data analysis, and new perspectives were proposed in this paper. Data loss was also more significant than anticipated, especially in regard to the measurements realized in Cameroon. However, it was possible to obtain, for the first time, some quantitative data for several players, and to lay the foundations for future research in this field. Moreover, without the use of an eye-tracker, it would have been impossible to collect accurate information on the precise interactions between the performers and their peers or with their audience: during the recordings, a video camera was systematically located in front of the face of the musicians, to monitor the entire process, and in many cases, the angle of the xylophonist’s head made it impossible to determine, for example, if the eyes were open or closed. In other terms, traditional techniques of observation and recording used by ethnomusicologists would not be sufficient for in-depth study of the eye-movements of Cameroonian players.

### Perspectives

This article is the result of a first foray into xylophone performance that hints at common practices and differences among xylophonists from different origins. Comparison between players from different cultural backgrounds needs to be further developed, to avoid a Western-centric approach, imperatively taking into account fundamental cultural differences: to truly compare practices between Western and Cameroonian performers, the nature of the musical material to be used in both cultures (or in any comparison between different cultures) is crucial. The conditions of performance of music (solo versus group, fixed versus improvised, isolated or with an audience, etc.) also need to be carefully designed.

The preliminary findings also open up the potential for future research on the pedagogy of percussion performance: what are the best practices to reach a high level of eye-hand synchronization? Is it possible to define profiles of individual performers that would ultimately lead to better tailored types of training – this training focusing on already existing skills, or on the contrary, proposing other approaches in gaze anticipation? Another fundamental question is linked to the notion of expertise, and longitudinal studies would provide invaluable data to better understand how experts develop their skills. For example, do experts develop automated processes? Are they able to better correct errors in eye-hand synchronization, or do they avoid building such errors better than non-experts during training? The fact that eye-tracking devices provide not only quantifiable data, but also qualitative data related to the nature of the gaze in its combination with musical gestures, will certainly encourage performers to use these types of tools to better understand the intimate nature of their relationship to musical performance.

### Ethics and Conflict of Interest

The author(s) declare(s) that the contents of the article are in agreement with the ethics described in http://biblio.unibe.ch/portale/elibrary/BOP/jemr/ethics.html and that there is no conflict of interest regarding the publication of this paper.

### Acknowledgements

This research was supported by 1) the Chaire Geste-Acoustique-Musique (GeAcMus), IDEX ‘Sorbonne Universités’ (ANR-11-IDEX-0004-02); 2) the Canadian Foundation for Innovation, Leaders opportunity Fund; 3) the Centre for Interdisciplinary Research in Music, Media and Technology (Montreal, Canada); 4) the Institut de Recherche et de Développement au Cameroun.

My sincere thanks to all of the musicians who participated in this study, to my translators and assistants in Cameroon, Valentin Angoni and Martin Mgbédié, to Catherine Massie-Laberge and John Sullivan for their assistance in data collection in Canada, and to my colleague Professor Catherine Guastavino for her help with statistical methods. I wish to thank Kristie Ibrahim for her editing skills, as well as Elke Lange and Lauren Fink for encouraging me to publish this article.
